# Cell-based therapy using miR-302-367 expressing cells represses glioblastoma growth

**DOI:** 10.1038/cddis.2017.117

**Published:** 2017-03-30

**Authors:** Mohamed Fareh, Fabien Almairac, Laurent Turchi, Fanny Burel-Vandenbos, Philippe Paquis, Denys Fontaine, Sandra Lacas-Gervais, Marie-Pierre Junier, Hervé Chneiweiss, Thierry Virolle

**Affiliations:** 1 Univ. Nice Sophia Antipolis, CNRS, Inserm, iBV, Nice 06108, France; 2 Service de Neurchirurgie, Hôpital Pasteur, CHU de Nice 06107, France; 3 Service d'Anatomopathologie, Hôpital Pasteur, CHU de Nice 06107, France; 4 Centre Commun de Microscopie Electronique Appliquée, Univ. Nice Sophia Antipolis, France; 5 CNRS UMR8246 Neuroscience Paris Seine - IBPS; Team Glial Plasticity; 7 quai Saint-Bernard 75005 Paris, France; 6 Inserm U1130, Neuroscience Paris Seine - IBPS; Team Glial Plasticity; 7 quai Saint-Bernard 75005 Paris, France; 7 University Pierre and Marie Curie UMCR18, Neuroscience Paris Seine - IBPS; Team Glial Plasticity; 7 quai Saint-Bernard 75005 Paris, France

## Abstract

Glioblastomas are incurable primary brain tumors that affect patients of all ages. The aggressiveness of this cancer has been attributed in part to the persistence of treatment-resistant glioblastoma stem-like cells. We have previously discovered the tumor-suppressor properties of the microRNA cluster miR-302-367, representing a potential treatment for glioblastoma. Here, we attempted to develop a cell-based therapy by taking advantage of the capability of glioma cells to secrete exosomes that enclose small RNA molecules. We engineered primary glioma cells to stably express the miR-302-367. Remarkably, these cells altered, in a paracrine-dependent manner, the expression of stemness markers, the proliferation and the tumorigenicity of neighboring glioblastoma cells. Further characterization of the secretome derived from miR-302-367 expressing cells showed that a large amount of miR-302-367 was enclosed in exosomes, which were internalized by the neighboring glioblastoma cells. This miR-302-367 cell-to-cell transfer resulted in the inhibition of its targets such as CXCR4/SDF1, SHH, cyclin D, cyclin A and E2F1. Orthotopic xenograft of miR-302-367-expressing cells together with glioblastoma stem-like cells efficiently altered the tumor development in mice brain.

Exosomes are extracellular nanoparticles of a size ranging from 30 to 120 nm which are secreted by most cell types in the human body.^1^ Exosomes are produced through the formation of multivesicular bodies (MVB) during the endosome maturation by the inward budding of the membrane.^2^ The sorting of biomolecules into exosomes involves specific protein machineries that ensure active and specific transport of functional mRNAs, miRNAs, proteins and metabolites.^3, 4, 5^ MVBs fuse with the cell membrane through a process, which depends on the release of exosomes out of the cells by Rab GTPase proteins. Considered to be the main route of excretion of harmful RNA and proteins for a long time,^2^ exosomes have been reconsidered instrumental for cell-to-cell communication, mediating the exchange of bio-molecules in normal and tumor tissues, including glioma.^6, 7^ Tumor-derived exosomes can promote tumor progression, metastasis^8, 9^ and immune system suppression by manipulating tumor microenvironment through reprograming the gene expression of the surrounding cells. The relative accessibility of tumor-derived exosomes from corporal fluids including blood, semen and urine led to the exosomes being used as diagnosis and prognosis tools in several cancers including hepatocellular carcinoma,^10^ gastrointestinal,^11^ lung cancers^12^ and glioma.^13^ The clinical interest in exosomes has been further strengthened by several studies, which describe exosome-based drug delivery strategies for anti-cancer treatment.^2, 14^ Numerous reports have suggested an important role of exosomes in glioma cell-to-cell communication and cell fate decision through the transfer of various molecules, including miRNA.^5, 15^

Glioblastomas (GBM) are the most common form of primary brain tumors which can affect adult patients of any age. These highly vascularized and infiltrating tumors are resistant to current treatment therapies and most often lead to a fatal outcome in less than 18 months. The current treatment involving radiotherapy and the use of temozolomide provides better results for patients presenting a methylated profile of MGMT gene.^16, 17^ However, the efficiency of this treatment, even involving the use of anti-angiogenic molecules (bevacizumab), is limited and this tumor remains incurable. The aggressive behavior of GBM, including resistance to current treatments and tumor recurrences, has been attributed to the presence of GBM stem-like or progenitor cells (GSC).^18, 19^ Thus, new treatment methods that specifically target GBM stem-like cells needs to be developed urgently in order to eradicate these incurable tumors.

Using a microRNA profiling approach in a collection of patient-derived primary culture of glioma stem-like cells (GSC), we have shown that the miR-302-367 cluster commits GSCs to an irreversible differentiated state and blocks their ability to initiate and progress tumors *in vivo*.^20^ In the present study, we show that patient-derived GSC, engineered to stably and constitutively express the miR-302-367cluster were able to release miR-302-367 containing exosomes. These exosomes were rapidly internalized by neighboring GSC, leading to the alteration of stemness and proliferative properties in a miR-302-367-dependent manner. Orthotopic xenograft of miR-302-367-expressing cells together with GSC efficiently altered the tumor development in mice brain, demonstrating their therapeutic potential for blocking tumor recurrences. Our study suggests that cell-based therapy could constitute an innovative anti-cancer solution.

## Results

### GSC expressing the miR-302 cluster represses SHH and SOX2 expression, proliferation and infiltration in a paracrine manner

**Figure 1:**
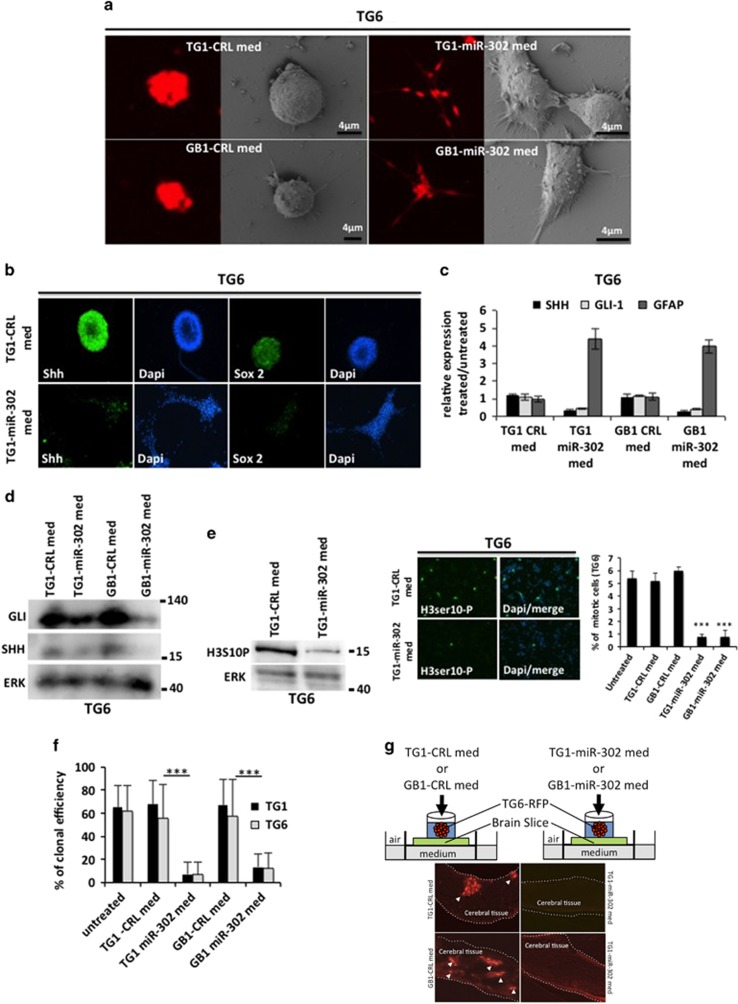
Conditioned medium harvested from miR-302 expressing cells repressed SHH, GLI1, SOX2 expression and naïve GSC proliferation and infiltration properties. (**a**) Naïve GSCs TG6 cells expressing RFP were treated with a conditioned medium harvested from TG1-CRL or GB1-CRL (left panel) or TG1-miR-302 or GB1-miR-302 (right panel). Fluorescence microscope and electron microscope were used to analyze the change in the morphology induced by conditioned media. (**b**) Immunofluorescence analysis of Shh and Sox2 expression level in naïve GSCs TG6 cells treated with control (TG1-CRL-med, upper panels) or miR-302 (TG1-miR-302-med, lower panel) conditioned media. (**c**) qRT-PCR analysis of SHH, GLI-1 and GFAP expression level in naïve TG6 GSCs cells treated with control (TG1-CRL-med or GB1-CRL-med) or miR-302 (TG1-miR-302-med, or GB1-miR-302-med) conditioned media. (**d**) Western blot analysis of SHH and GLI-1 expression level in naïve TG6 GSCs cells treated with control (TG1-CRL-med or GB1-CRL-med) or miR-302 (TG1-miR-302-med, or GB1-miR-302-med) conditioned media. (**e**) Western blot (left picture) and immunofluorescence (right picture) analysis of Histone-H3 phosphorylation (marker of mitosis) in naïve GSCs TG6 treated with control (TG1-CRL-med or GB1-CRL-med) or miR-302 (TG1-miR-302-med, or GB1-miR-302-med) conditioned media. The histogram (right) displays a quantification of mitotic GSCs naïve cells in both conditions. (**f**) Clonal proliferation of naïve GSCs cells (TG1 or TG6) treated with control (TG1-CRL-med or GB1-CRL-med) or miR-302 (TG1-miR-302-med, or GB1-miR-302-med) conditioned media. (**g**) Naïve GSCs TG6 cells stably expressing red fluorescent protein (RFP) were seeded on the top surface of an organotypic mouse brain slice. The organotypic cultures were treated with control (TG1-CRL-med or GB1-CRL-med) or miR-302 (TG1-miR-302-med, or GB1-miR-302-med) conditioned media. After 3 weeks, each organotypic culture was embedded in paraffin and sliced. Cell infiltration and growth within the neural host tissue were visualized by tracking the red fluorescent protein. Dashed lines mark the neural tissue boundaries

To assess whether GBM cells expressing the miR-302-367 cluster might affect the stemness state of naïve GSC in a paracrine manner, we harvested conditioned media from two different GSC primary cultures stably expressing the miR-302-367 cluster (TG1-miR-302 and GB1-miR-302) or a non-relevant construct (TG1-CRL and GB1-CRL). We then treated a control GSC primary culture (stably expressing the red fluorescent protein, TG6-red fluorescent protein (RFP)) with TG1-miR-302 and GB1-miR-302 conditioned media and tracked the evolution of cell morphology over 4 days. While the cells incubated in the control medium remained unchanged, the cells incubated in the medium collected from miR-302-367 expressing cells became adherent and displayed a typical morphology of more differentiated cells with low nuclear-cytoplasmic ratio and long cell extension (Figure 1a). These phenotypic changes occurred together with repression of self-renewal markers such as SOX2 and SHH, as well as a clear increase in GFAP expression (Figures 1b–d). The treatment of naïve GSC with miR-302 conditioned medium strongly repressed the number of mitotic cells and impaired their ability to be clonal (Figures 1e and f). To assess the effects of miR-302 conditioned medium on GSC ability to infiltrate a cerebral tissue, we used organotypic cultures of mouse brain slices (MBSs). We placed cloning rings on MBSs surface (Figure 1g) in which we have seeded naïve TG6-RFP cells for 3 weeks. The seeded cells were regularly treated with either control or miR-302 conditioned medium. While TG6 cells, in the control conditions, confirmed their strong ability to invade and survive within the cerebral tissue,^20^ the treatment with miR-302 conditioned medium repressed their ability to penetrate cerebral tissues. Taken together, these results show that GSCs expressing the miR-302 cluster secrete molecules that efficiently altered the stemness state and repressed cell proliferation and tissue infiltration.

### TG1-miR-302 and GB1-miR-302 cells secrete exosomes capable to repress GSC stemness and proliferation

**Figure 2:**
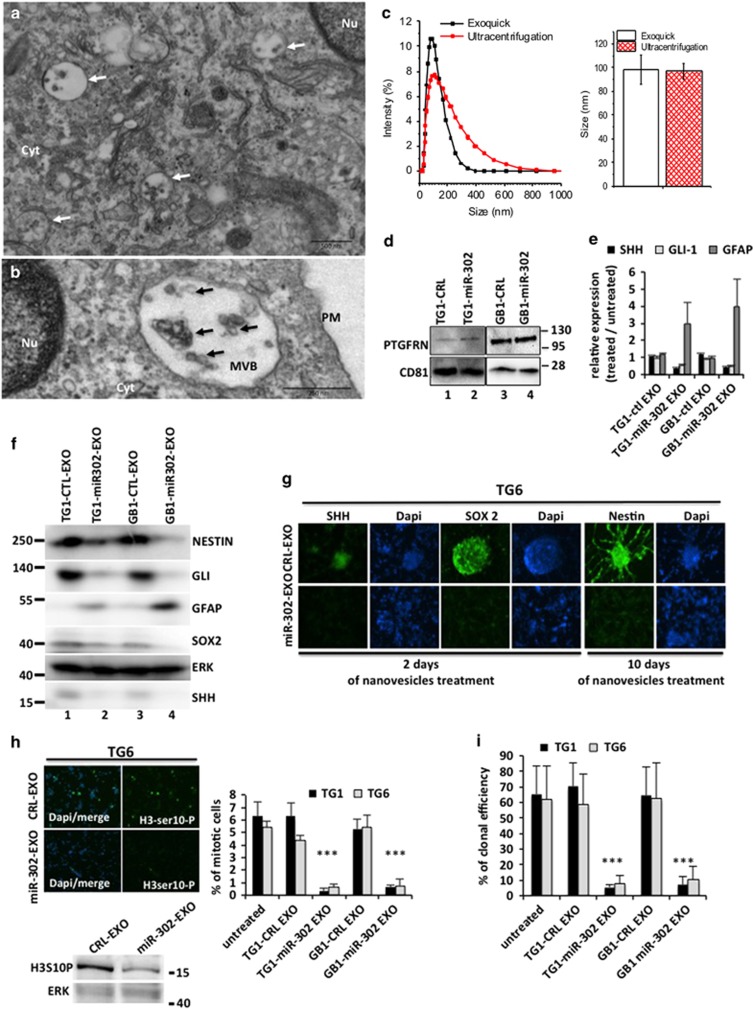
TG1-miR-302 and GB1-miR-302 cells secrete exosomes capable to repress GSC stemness and proliferation. (**a**,**b**) Electron micrographs showing exosomes within multi-vesicular bodies (MVB) of TG1-miR-302 cells. (**c**) Comparison of size distributions of exosomes isolated by Exoquick or ultracentrifugation as measured by DLS. (left) DLS percent intensity by size (nm) for Exoquick (black line) or ultracentrifugation (red line). (right) Mean±SD particle diameter for Exoquick (black) and ultracentrifugation (dashed red) as measured by DLS, (*n*=6). (**d**) Protein extracts prepared from TG1-CRL and GB1-CRL exosomes (lanes 1 and 3 respectively) or TG1-miR-302 and GB1-miR-302 exosomes (lanes 2 and 4 respectively). Exosomal proteins were electrophoretically separated on 4–12% SDS-PAGE gel and analyzed by western blotting using antibodies against PTGFRN and CD81. (**e**) Naïve GSCs TG6 cells were treated with exosomes purified from TG1/GB1 control cells or from TG1/GB1 miR-302 expressing cells. The expression level of SHH, Gli-1 and GFAP in the exosomes treated cells was quantified by qRT-PCR. (**f**) Western blot analysis of NESTIN, GLI-1, GFAP, SOX2, SHH (ERK as loading control) following 10 days of control exosomes (CRL-EXO) or miR-302 exosomes (miR-302-EXO) purified from TG1 (lanes 1 and 2) or GB1 (lanes 3 and 4) cells. (**g**) Immunofluorescence analysis of SHH, SOX2 and NESTIN expression level in naive GSCs (TG6) treated with control exosomes (CRL-EXO) or miR-302 exosomes (miR-302-EXO) purified from TG1-miR-302. (**h**) western blot (lower) and immunofluorescence (upper) analysis of Histone-H3 phosphorylation (marker of mitosis) in naïve GSCs (TG6) treated with control exosomes (CRL-EXO) or miR-302 exosomes (miR-302-EXO) purified from TG1-miR-302 or GB1-miR-302 cells. The histogram (right) displays the quantification of mitotic TG1 or TG6 naïve cells. (**i**) Clonal proliferation of naïve GSCs cells (TG1 and TG6) treated with control exosomes (CRL-EXO) or miR-302 exosomes (miR-302-EXO) purified from TG1-miR-302 or GB1-miR-302

To identify the molecules responsible for the anti-tumor effect we performed size-based ultrafiltration of the conditioned media. We noticed that a fraction with high molecular weight (<100 kDa) exclusively retained the anti-tumor effect (data not shown). We hypothesized that exosomes might mediate the observed paracrine effect. Electron microscopy analysis of TG1 cells showed the presence of several MVB in the cytoplasm, harboring typical cup-shaped small vesicles of 30–70 nm in size (Figures 2a and b). We purified the nanovesicles from conditioned media of either TG1 and GB1-CRL or TG1-miR-302 and GB1-miR-302 cells using Exoquick kit. Dynamic light scattering (DLS) measurements confirmed the presence of nanovesicles in the conditioned media with typical size of~100 nm in majority (Figure 2c). Similar nanovesicle sizes were obtained by ultracentrifugation (Figure 2c). Protein extraction from these nanovesicles revealed expression of exosome markers such as the tetraspanin CD81 or the prostaglandin F2 receptor negative regulator (PTGFRN) (Figure 2d). We then used exosomes suspended in a fresh medium (NS34+) for treating naïve self-renewing TG6. After treatment the cells displayed a clear increase of GFAP expression and the repression of stem/progenitor markers such as SHH, SOX2 after 2 days and Nestin after 10 days following the addition of TG1-miR-302 or GB1-miR-302-derived exosomes (miR-302-EXO) (Figures 2e–g; Table 1). Furthermore, miR-302-EXO treatment induced a drastic decrease in the number of mitotic cells and the capacity of TG1 and TG6 cells to be clonal (Figures 2h and i). The control exosomes (CRL-EXO) isolated from TG1-CRL or GB1-CRL did not modify cell behavior and proliferation or the expression of stemness markers.

### The miR-302 cluster is transferred to neighboring cells via the secretion of exosomes

**Figure 3:**
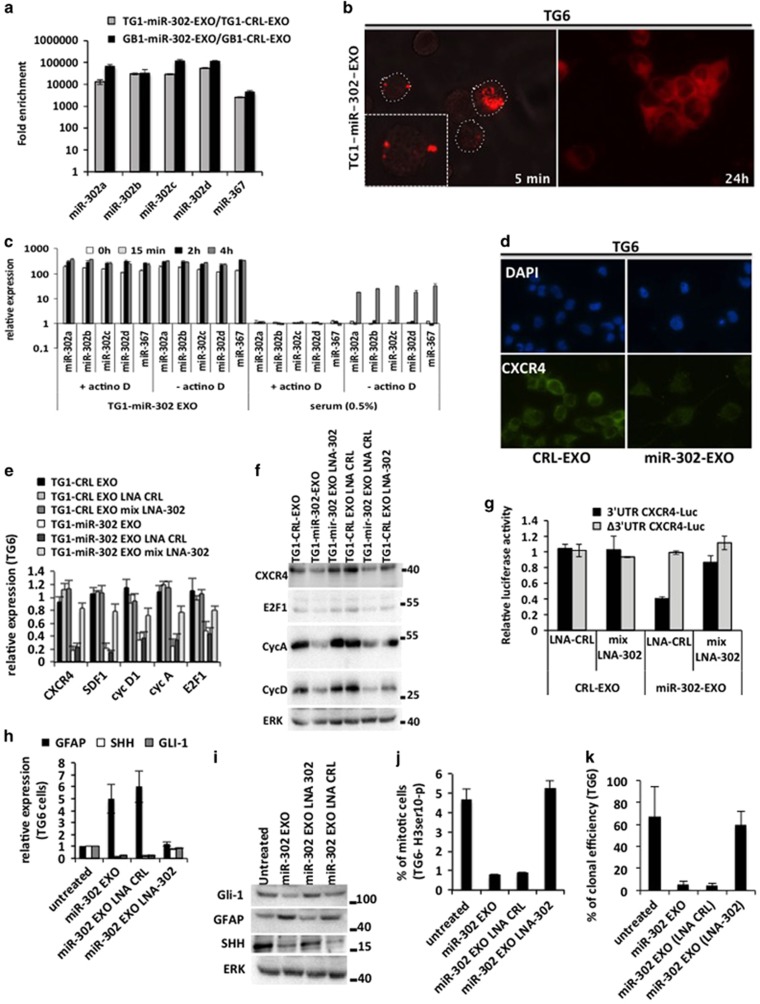
Secreted exosomes transferred functional miR-302-367 cluster to neighboring cells. (**a**) q-RT-PCR analysis of cluster miR-302 expression levels in the exosomes derived from miR-302 cells (TG1-miR-302-EXO and GB1-miR-302-EXO). Data are normalized using RNA SNORD54 (U54) as internal control. The results are expressed as a ratio between the relative miR expression found in miR-302 EXO and in CRL EXO, according to the ΔΔCT method. (**b**) Naïve TG6 were treated with Dye-labeled exosomes extracted from TG1-miR-302 (pre-labeled with DiO16 membrane-dye). Fluorescence microscope was used to track exosome fusion at 5 min and 24 h. The dash circle surrounds one single cell. (**c**) Time course analysis of miR-302-367 cluster member expression following exosomes internalization by the recipient TG6 cells. The Actinomycin D was used to block RNA synthesis (endogenous expression of cluster miR-302) in exosome (TG1-miR-302-EXO) or serum (0.5%) treated naïve TG6. Data are normalized using RNA SNORD54 (U54) as internal control. The results are expressed as a ratio against the time 0 h. (**d**) Immunofluorescence analysis of CXCR4 in recipient TG6 cells treated with control exosomes (TG1-CRL-EXO) or exosomes purified from miR-302 cells (TG1-miR-302-EXO). (**e**) q-RT-PCR analysis of the expression level of CXCR4, SDF1, CycD1, CyCA and E2F1 transcripts in recipient TG6 cells treated with control exosomes (TG1-CRL-EXO) or exosomes purified from miR-302 cells (TG1-miR-302-EXO). A mixture of LNA probes specific to each miR-302 members (LNA-302) were used to neutralize the miR-302 effects. Scramble LNA sequences were used as negative control (LNA-CRL). For each condition, gene expressions have been normalized using GAPDH as internal control. The results have been presented as a ratio against untreated condition. (**f**) Western blot showing the expression level of CXCR4, E2F1, CycA, CycD1, ERK (loading control) in recipient TG6 cells treated with control exosomes (TG1-CRL-EXO) or exosomes purified from miR-302 cells (TG1-miR-302-EXO). A mixture of LNA probes specific to each miR-302 members (LNA-302) were used to neutralize the miR-302 effects. Scramble LNA sequences were used as negative control (LNA-CRL). (**g**) Dual-luciferase assay of 293 T HEK cells transfected with luciferase reporter constructs containing CXCR4 3'UTR fused to the luciferase gene (3′UTR CXCR4-Luc) or identical construct but deleted of miR-302 recognition elements (Δ3′UTR CXCR4-Luc). The cells were simultaneously transfected with mix LNA-302 or LNA-CRL. The cells were treated with control exosomes (TG1-CRL-EXO) or exosomes purified from miR-302 cells (TG1-miR-302-EXO). (**h**–**j**) TG6 cells were untreated or treated with TG1-miR-302-EXO in presence or absence of LNA-CRL or LNA-302. (**h**) q-RT-PCR (For each condition, gene expressions have been normalized using GAPDH as internal control) and (**i**) western blot analysis of the expression level of GFAP, SHH and GLI-1 transcripts and protein respectively in recipient TG6 cells 24 h after treatment. (**j**) Quantification of immunofluorescence analysis of Histone 3-phospho-Ser 10 shows the percentage of mitotic TG6 cells 24 h after treatment. (**k**) Limiting dilution of naïve TG6 cells to assess their clonal proliferation capacity

Following the exosome extraction from TG1 or GB1 conditioned media, we have assessed the presence of the miR-302 cluster in both the exosome pellet and the supernatant. Each member of the miR-302 cluster has been strongly detected by qRT-PCR in miR-302-EXO while weakly detected in the corresponding supernatant and undetected in similar preparation from TG1-CRL or GB1-CRL (CRL-EXO) (Figure 3a and Table 2). To assess the interactions between the exosomes and the recipient cells, we labeled TG1-miR-302-EXO with fluorescent lipophilic carbocyanine diIC16 and incubated naïve TG6 cells with the resulting fluorescent exosomes. The cells were then extensively washed before the fluorescence analysis. The images obtained using a confocal fluorescence microscope revealed a physical interaction between the fluorescent exosomes (red fluorescence) and single naïve TG6 cells (unlabeled) within the first 5 min following the treatment with red-fluorescent exosomes (Figure 3b, *left*). Twenty-four hours after treatment, we observed the transfer of fluorescence to the plasma membrane of TG6 recipient cells, indicating the internalization of the fluorescent exosomes (Figure 3b, *right*). Lysis of the TG6 recipient cells followed by qRT-PCR analysis revealed a strong increase in the expression level of all microRNAs members of miR-302 cluster. The enrichment of recipient cells with the cluster miR-302 occurred within 15 min following the treatment with exosomes. In comparison, cells treated with CRL-EXO did not show any enrichment with the cluster miR-302. The rapid enrichment in the cluster miR-302 (15 min) indicates an external source of these microRNAs rather than internal neo-synthesis. We have previously shown that miR-302 cluster neo-synthesis occurred few hours later after serum induction in GSC.^20^ Four hours of serum stimulation induced 20-fold enrichment with the endogenous miR-302b. In comparison, 300-fold enrichment was obtained after only 15 min of miR-302-Exo treatment (Figure 3c and Supplementary Figure 1a). To demonstrate that miR-302 expression in recipient TG6 cells was the result of the transfer from exosomes as opposed to endogenous synthesis, we inhibited the transcription by blocking the RNA polymerase II using actinomycin D. The blockade of the polymerase did not prevent the enrichment in miR-302 cluster in the recipient cells treated with miR-302-EXO, while it efficiently blocked the endogenous synthesis induced by the serum (Figure 3c). These results indicate that exosomes carrying miR-302 are able to rapidly deliver a high quantity of miR-302 cluster to the recipient cells. As a result, the known targets of the mir-302 cluster such as CXCR4, SDF1, Cycline D1, Cycline A and E2F1^20, 21, 22, 23^ were repressed in TG6 cells upon TG1-miR-302-EXO treatment (Figure 3d–f). To probe the direct targeting of the 3′ untranslated region (3′UTR) of CXCR4 by the exosomal miR-302, we employed a luciferase reporter assay. The luciferase activity of a reporter construct, which contained the 3′UTR of CXCR4 fused to the 3′ end of the luciferase coding sequence (Δ3′UTR CXCR4-Luc), was significantly reduced when the cells were treated with miR-302-EXO. A similar construct lacking miR-302 binding elements (Δ3′UTR CXCR4-Luc) remained insensitive to the miR-302-EXO treatment (Figure 3g). Supplementary Figure 2 showed that CXCR4 3′UTR was efficiently targeted by miR-302a, b, c, d but not by miR-367. Transfection of recipient TG6 cells with a mixture of Locked Nucleic Acid (LNA) probes, which specifically target the miR-302 cluster (mix LNA-302) (Supplementary Figure 1b), suppressed the miR-302-EXO effects, preventing the invalidation of the miR-302 targets (Figures 3e–g), increase of GFAP, repression of SHH pathway (Figures 3h and i) as well as the decrease of cell proliferation and clonal efficiency (Figures 3j and k). Taken together, these results show that delivery of the miR-302 cluster is required to alter the stemness and proliferation properties of GSC.

### Expression of the miR-302 cluster in GSC promotes a paracrine tumor suppressor effect *in vivo*

**Figure 4:**
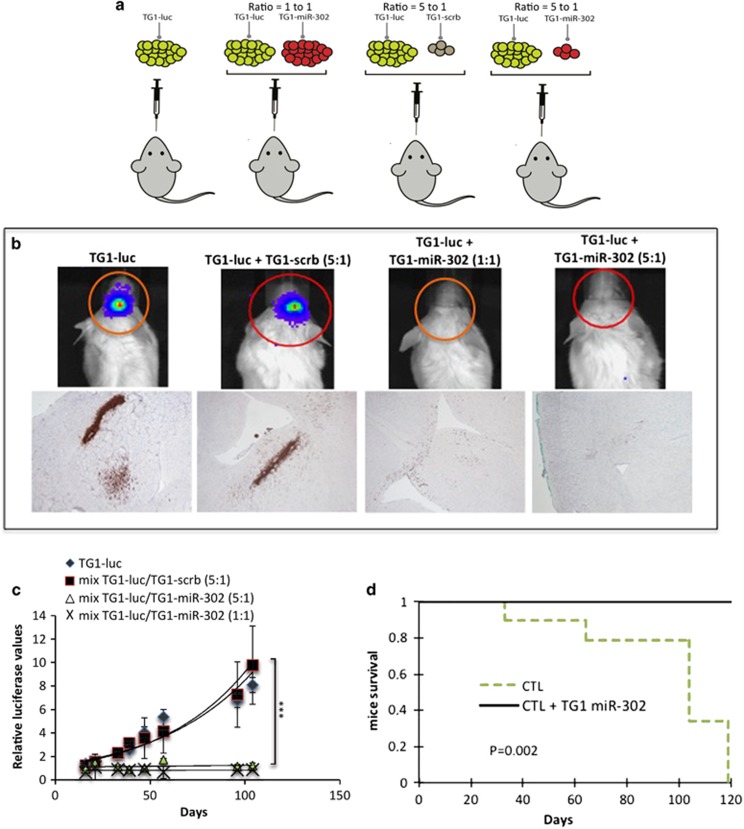
The co-injection of miR-302 overexpressing cells along with naïve GSC inhibits tumor development in mouse brain. (**a**) Cartoon representing the procedure of injection. In all, 200 000 TG1 control cells stably expressing the luciferase gene (TG1-luc) have been injected into the striatum of male NOD.CB17-Prkdcscid/NCrHsd mice, alone or in combination with 40 000 TG1 stably expressing a scramble construct (TG1-scrb) or 40 000 TG1 stably expressing the miR-302-367 cluster (TG1-miR-302) (ratio= 5 to 1 respectively) or 200 000 TG1-miR-302 (ratio=1 to 1). (**b**) Live imaging has been used to analyze tumor formation and growth in living animals (IVIS lumina II) for the procedures of co-injections (top panels). After killing the animals, the brains have been dissected, embedded in paraffin and sliced in order to undergo a staining using specific human alpha vimentin to visualize the human tumor cells (lower panels). (**c**) Quantification of tumor growth in living animals (IVIS lumina II). The results showed significant differences (*P* value <0.01, *t*-test) between the group of control cells (TG1-luc and TG1-luc mixed with TG1-scrb cells) and the group of TG1-luc mixed with TG1-miR-302 (ratio=1 to 1 and 5 to 1). (**d**) Mice survival analysis using Kaplan–Meier plot. The two groups correspond to 10 mice that have undergone intracranial injection of TG1 control cells alone or mixed with TG1-scrb (5 mice per conditions, *n*=10) and TG1 control cells mixed with TG1 miR-302 cells at a ratio of 1 to 1 or 5 to 1 respectively (5 mice per conditions, *n*=10). *P*-value=0.002 (Wilcoxon test)

GBM are most often surgically removed. However and despite aggressive treatments, tumor initiation and growth recurs and this is a cause of high mortality of the disease. In this context, we sought to assess whether the paracrine effect of miR-302 might act as a tumor suppressor *in vivo* by blocking tumor initiation and growth. We have conducted orthotopic xenograft experiments by performing stereotaxic injections of TG1-luc (TG1 cells stably expressing luciferase) in the striatum of four groups of immuno-deficient NOD/SCID mice. TG1-luc cells were injected alone as a control of tumor formation, together with TG1-miR-302 cells (at a ratio of one-to-one and five-to-one respectively) or together with TG1 cells expressing a non-relevant construct (TG1-scrb, control) (Figure 4a). Live imaging using the luciferase activity of TG1-luc cells allowed monitoring tumor growth over time. Our results showed that TG1-luc alone or mixed with TG1-scrb were capable of initiating and developing tumors *in vivo* (Figures 4b and c). Immuno-staining with a human-specific anti-vimentin antibody revealed the presence of infiltrating cells and the formation of several tumor foci in the striatum of the control animals (Figure 4b). However one-to-one as well as five-to-one ratio between TG1-luc and TG1-miR-302 cells altered tumor initiation, growth and the formation of infiltration foci (Figures 4b and c). In addition, our data indicate that a minor population of TG1-miR-302 cells (one TG1-miR-302 for five TG1-luc) was sufficient to suppress tumor development *in vivo* (Figures 4b and c). In these conditions, the immuno-staining revealed the infiltration of the striatum and the corpus callosum by isolated tumor cells and the absence of massive proliferation and infiltration, as observed in the brain of the control animals (Figure 4b). Accordingly, TG1-miR-302 co-injection maintained mice survival while mice in the control group died within 100 days (Figure 4d).

These results indicate that miR-302 over-expression in GSC induced an efficient paracrine tumor suppressor effect on neighboring tumor cells.

## Discussion

Numerous reports have confirmed the tumor suppressor effect of the miR-302-367 in a variety of cancers^20, 24, 25, 26, 27^ suggesting its potential use for therapeutic applications. In this context, efficient delivery of therapeutic small RNAs constitutes the main obstacle to make small interfering RNA available for clinical use. In this study, we took advantage of the capacity of GSC to abundantly secrete exosomes^13^ to create patient-derived cells able to continuously deliver exosomes enclosing the miR-302-367 cluster. The secretion of these exosomes ensures the propagation of the miR-302 effects to neighboring naïve GSC. Once in the recipient cells, the miR-302-367 cluster efficiently represses its targets, namely Cyclin D1, Cyclin A, E2F1 and the CXCR4 pathway.^20^ The consequences of this repression were reflected by a strong repression of GSC mitosis, clonal proliferation, as well as the capacity of GSC to penetrate and proliferate within a cerebral host tissue. These effects were drastically inhibited when the miR-302-367 cluster expression was antagonized in the recipient cells by the use of specific LNA-302 probes. Importantly, although GSCs release vesicles of all sizes in their media, the nano-sized ones are efficient in promoting the miR-302-367 effects, since conditioned media depleted of small vesicles lost their capacity to induce the miR-302 effects on GSC (data not shown).

In light of the idea that nanoparticles are capable of influencing normal and tumor cells in the tumor microenvironment,^28, 29, 30^ our results show that the miR-302-367 exosomes efficiently changed the cellular behavior, by compromising stemness properties and inhibiting tumor development *in vivo*. Our data strengthen the choice of using the strategy of delivering miRNA or anti-miR by exosomes as a therapeutic option, which has been recently highlighted by studies showing that exosomal delivery of anti-miR-9 or miR-1, efficiently modifies GBM cell chemosensitivity,^31^ migration and proliferation.^15^ Considering the therapeutic efficiency, one critical aspect of this strategy is to achieve an efficient and prolonged delivery of exosomes into the organism. Akao *et al.* have shown that intravenous THP1-cells injection in nude mice, pretransfected with miR-143, induced an increased level of miR-143 in serum and kidneys,^32^ demonstrating efficient delivery of miRNA into the organism. Based on these studies, we have chosen to perform the co-transplantation of patient-derived-GSCs expressing the tumor-suppressor cluster miR-302-367 along with control GSCs, into the brain of immunodeficient mice, rather than making injections of exosomes, which would have provided only transitory effects. This strategy was particularly efficient since it induced a long-term inhibition of not only tumor initiation and development but also tumor growth of already established tumors. This tumor suppressor effect is of particular interest, in term of therapeutic applications, to prevent tumor relapse and to inhibit tumor growth in patients unable to undergo surgical resections.

The results of this approach that we obtained in mice are a proof-of-principle that cell-based therapy for cancer treatment deserves to be considered. Besides the GSC, other exosome-secreting cells such as macrophages can be isolated from patients and genetically modified to stably express therapeutic RNA molecules for tumor treatment.

The transplantation of these cells directly into the tumor site during the surgery would allow extended delivery of tumor suppressor exosomes locally, avoiding heavy and stressful periodic injections of exosomes to the patient.

In conclusion, we believe that our study will spark interest in using cell-based therapy for *in situ* delivery of therapeutic exosomes to target human GBM.

## Materials and methods

### Electron microscopy

Scanning electron microscopy (SEM): For SEM analysis, cells were fixed with 1.6% glutaraldehyde in 0.1 M phosphate buffer for several times before washing with distilled water, and cryoprotection in a solution of 30% glycerol for 1 h. The samples were then dehydrated in a graded ethanol series, and finally immersed in hexamethyldisilazane (Carl Roth, Karlsruhe, Germany), and dried at room temperature. Sample were then mounted on aluminium stubs and sputter-coated with a 3-nm gold–palladium coating (Cressington 308EM, UK) prior to analysis with a Field Emission Scanning Electron Microscope (FESEM JEOL 6700F, Japan). Transmission Electron Microscopy (TEM): For TEM, the samples were fixed by the same procedure as for SEM. After an adequate fixation, the samples were rinsed in 0.1 M cacodylate buffer, and post-fixed for 1 h in 1% osmium tetroxide in 0.1 M cacodylate buffer. Samples were then rinsed in distilled water, dehydrated in alcohols and lastly embedded in epoxy resin. Contrasted ultrathin sections (70 nm) were analyzed under a JEOL 1400 transmission electron microscope mounted with a Morada Olympus CCD camera.

### Dynamic light scattering (DLS)

DLS measurement was performed with a Zetasizer Nano-ZS (Malvern Instruments, Malvern, UK). Samples were diluted in 100 *μ*l of 1 × PBS. 3 × 12 measurement runs were performed using the standard settings (Refractive Index=1.33, temperature=25 °C, viscosity=0.8882 and dielectric constant=79.0). The results were plotted using OriginPro 2015.

### Microvesicles extraction

~10^6^/ml GSCs were cultured in a defined microvesicle-free medium (NS34+ medium, Fareh *et al.*). After 72 h, the GSCs were pulled down by centrifugation (276 × *g*, 4 °C, for 5 min) and the conditioned medium was collected, filtered through a 0.2-*μ*m filter (Millipore, Fontenay sous Bois, France) and concentrated using 100 kDa cut-off Amicon Ultra centrifugal filter (Millipore,). The concentrated conditioned medium was used to purify the microvesicles by ultracentrifugation at 200 000 × *g* for 110 min.

### Reagents and antibodies

Cell culture reagents including DMEM, F12, glutamine, Hepes, Sodium bicarbonate, N2, G5, and B27, pENTR cloning kit, LR Clonase II, Superscript II reverse transcriptase, DiIC16(3), DiO16(3) and TRIzol Reagent were purchased from Life technologies (Cergy Pontolse, France). Fetal calf serum (FCS) was purchased from Hyclone (Brebières, France), and Exoquick kit was purchased from Ozyme (St. Quentin en Yvelines, France). Hoechst 33342 and Actinomycin D were supplied by Sigma (St. Quentin Fallavier, France), Micromount Mounting Media was purchased from Leica Biosystems (Nanterre, France). Taqman Reverse transcription microRNA kit, Universal Taqman PCR Master Mix, and Taqman probes were purchased from Applied Biosystems (Villebon sur Yvette, France). Enhanced chemiluminescence detection reagent was purchased from Bio-Rad (Marnes la Coquette, France).

The antibodies used in this study are listed here: Goat polyclonal anti-Shh (1/50°, sc1194, Santa Cruz Biotechnology, Santa Cruz, Germany); Mouse monoclonal anti-CXCR4 (1/50°, MAB 172, R&D Systems, UK); Rabbit polyclonal anti-H3-phospho-S10 (1/100° ab5176, Abcam, Paris, France); Rabbit polyclonal Anti-sox 2 (1/100° Sox-2 (H65), sc-20088, Santa Cruz Biotechnology, Inc); Mouse monoclonal Anti-Nestin (1/100°, ab22035, Abcam); Anti-goat Alexa 488 (1/500° Dusseldorf, Germany); Anti-rabbit Alexa 488 (1/500°, Dusseldorf, Germany); Anti-mouse Alexa 488 (1/500°, Dusseldorf, Germany); Anti-goat Alexa 546 (1/500°, Dusseldorf, Germany); Anti-rabbit Alexa 546 (1/500°, Dusseldorf, Germany); Anti-mouse Alexa 546 (1/500° Dusseldorf, Germany); Anti-GFAP (1/200°, 2203PGF, EUROPROXIMA, Arnhem, The Netherlands). Anti-PTGFRN (1/500°, ab174180, abcam); Anti-CD81 (1/500°, Clone # 454720, R&D systems, Abingdon).

The mix miRCURY LNA Knockdown probes that target miR-302a, miR-302b, miR-302c and miR-302d were purchased from EXIQON (Madrid, Spain). Scrambled sequence was used as a control.

### Cell culture

The GSCs primary cell lines TG1, TG6 and GB1 were isolated from human GBM as described elsewhere.^20, 33, 34^ When kept as self-renewing GSCs, neurospheres were grown in NS34+ medium containing EGF and bFGF (DMEM-F12 1/1 ratio, 10 mM glutamine, 10 mM Hepes, 0.025% sodium bicarbonate, N2, G5, and B27). The medium for cell differentiation (MFCS) was composed of DMEM-F12, glutamine 10 mM, Hepes 10 mM, 0.025% sodium bicarbonate, and 0.5% FCS. In the experiments of differentiation, the neurospheres were dissociated and 500 000 single cells were cultured in MFCS.

### Plasmid constructs and stable cell lines

The miR-302 cluster was amplified from genomic human DNA by PCR (Forward primer: 5′-GGCTGAAGTCCCTGCCTTTTACCC-3′, Reverse primer: 5′-TGGCTTAACAATCCATCACCATTGC-3′) and cloned into a pENTR commercial vector (life technology). Subcloning in the 2K7 blasticidin lentiviral vector (2K7BSD) was realized by recombination in the presence of LR clonase II. A scramble form of shLuc (2K7BSD-shLuc-scb) that prevents inhibition of the luciferase gene was used as non-relevant construct (CRL). Lentiviral particles were produced by transfecting the 293 T cell line with the 2K7BSD-Cluster mir-302 or 2K7BSD-shLuc-scb constructs along with the packaging vectors (Invitrogen, Waltham, USA). After lentiviral infection, cell lines stably expressing the miR-302 cluster (TG1 cluster miR-302 and GB1 cluster miR-302) or the control shLuc-scb (TG1-CRL and GB1-CRL) were selected in blasticidin containing medium (1 *μ*g/ml) for 15 days. Two stable cell lines were developed from independent viral productions/infections and exhibited similar behaviors. Cells stably expressing the RFP were obtained after the infection of GSCs with 2K7BSD-RFP lentiviral particles.

### Immunofluorescence

Cells were grown on poly-L-lysine-coated glass coverslips in NBE, MFCS, or conditioned mediums. At indicated time points, cells were fixed with methanol 10 min at −20 °C, and washed with pre-chilled PBS twice. Blocking and antibody hybridization were performed in PBS containing 10% FCS and 0.1% Triton × 100. After 1 h of incubation with the primary antibodies at room temperature, cells were washed three times with PBS and stained for 30 min at room temperature with species-specific fluorophore-coupled secondary antibodies. At the same time, nuclei were stained with Hoechst 33342 (1 *μ*g/ml). The slides were washed twice with PBS, once with distilled water, and finally mounted with Gel Mount solution. Immunofluorescence and transmission light pictures were taken with a Nikon eclipse Ti microscope (Nikon, Champigny sur Marne, France).

### Clonogenical assay

Neurospheres were dissociated by 20 times gentle pipetting up and down to obtain single cells. Overall, 10 cells were seeded in each well of 96-well plates containing control or miR-302 conditioned medium. After 21 days of incubation, each well was examined and the number of neutrospheres was counted. The experiments were repeated three independent times.

### Quantitative real-time RT-PCR

RNA was extracted using Trizol reagent. MicroRNA and mRNA expression levels were quantified by two step real-time RT-PCR. Reverse transcription steps were performed with Superscript II reverse transcriptase and Taqman reverse transcription microRNA kit for mRNA and miRNA, respectively, following the manufacturer's instructions. Real-time PCR experiments were performed using universal Taqman PCR Master Mix. Small nucleolar RNA SNORD54 (or U54) and GAPDH expression was used as internal control to normalize gene expression. Fold changes were estimated against control conditions using the ΔΔCT method.

### Orthotopic xenografts

2.10^5^ TG1 control cells stably expressing luciferase (TG1-luc) were resuspended in 5 *μ*l of Hanks balanced salt solution (Invitrogen) and stereotactically implanted unilaterally into the striatum of male NOD.CB17-Prkdcscid/NCrHsd mice (Harlan, France). Luciferase expressing cells were co-injected with TG1 cells stably expressing scrambled sequence of the cluster miR-302 (TG1-scrb) or stably expressing the cluster miR-302. The luciferase activity allowed the monitoring of tumor development in living animals. Cell survival and tumor growth were monitored and quantified in the living animals up to 120 days by detecting the luciferase activity with the IVIS Lumina II system (Caliper Life Sciences, Hopkinton, MA, USA).

### Organotypic MBS culture

Brains were dissected from new born mice, embedded in 4% agar-agar artificial cerebrospinal fluid (124 mM NaCl, 3 mM KCl, 26 mM NaHCO_3_, 2 mM CaCl_2_, 1 mM MgSO_4_, 1.25 mM KH_2_PO_4_, 10 mM glucose), and cut in slices of 400 mm thickness using a vibratome. The slices were placed on a Millicell-CM (0.4 *μ*m) culture plate, inserted and maintained under air–liquid interface conditions for more than 3 weeks.

### Paraffin embedding

Mice brain slides were fixed with 4% paraformaldehyde for 20 min at room temperature, then washed with PBS. Samples were subsequently dehydrated with the following sequence of incubations: ethanol 70% 15 min, twice; ethanol 90% 15 min; ethanol 95% 15 min; ethanol 100% 5 min, thrice; Roticlear 5 min.

### *Ex vivo* tumorigenesis assay

Ten neurospheres from TG1-CRL and TG1-miR-302 cells stably expressing the RFP were seeded at the top surface of the MBS and cultured under air–liquid interface conditions for 3 weeks. The cells were treated with the conditioned mediums every 48 h. The cell infiltration and growth was visualized within the thin slices by tracking the RFP signal using a Nikon eclipse Ti microscope.

## Figures and Tables

**Table 1:** Quantification of SHH, SOX2, NESTING immunofluorescence in naïve TG6 and TG1 treated during 2 days and 10 days by exosomes purified from TG1/GB1-CRL or TG1/GB1-miR-302

	**TG6**			**TG1**		
	**Days of treatment**			**Days of treatment**		
	**2 days**		**10 days**	**2 days**		**10 days**
	**Shh**	**SOX2**	**Nestin**	**Shh**	**SOX2**	**Nestin**
*TG1-CRL*						
med	90.38% ±3.8	94.68% ±0.73	99.78% ±0.28	91.86% ±4.53	93.18% ±5.02	99.4% ±0.75
EXO	91.80% ±2.99	94.48% ±1.45	97.77% ±2.96	90.08% ±4.95	92.03% ±0.67	100% ±0.00
						
*TG1-miR-302*						
med	8.32% ±2.78	7.32% ±1.50	1.21% ±0.62	7.79% ±1.54	6.81% ±2.56	0.14% ±0.18
EXO	9.06% ±1.6	6.24% ±1.34	0.65% ±0.23	11.17% ±4.03	6.07% ±0.86	1.00% ±0.44
						
*GB1-CRL*						
med	97.19% ±1.16	91.4% ±5.15	98.78% ±0.80	89.12% ±3.37	93.57% ±5.14	100% ±0.00
EXO	94.30% ±1.83	96.43% ±0.46	99.11% ±1.17	95.89% ±1.41	95.50% ±1.97	99.72% ±0.36
						
*GB1-miR-302*						
med	8.68% ±1.84	8.51% ±1.32	0.89% ±0.59	10.70% ±4.15	5.79% ±1.30	0.77% ±0.32
EXO	8.40% ±1.59	6.20% ±1.53	0.26% ±0.35	8.16% ±0.79	7.10% ±1.10	0.15% ±0.20

One hundred cells have been counted in three separate experiments

**Table 2:** Quantification of miR-302-367 expression by q-RT-PCR in the supernatant and the pellet of exosome preparations from TG1/GB1-CRL or TG1/GB1-miR-302

	**TG1-CRL**		**TG1-miR-302**		**GB1-CRL**		**GB1-miR-302**	
	**Supernatant**	**Pellet (EXO)**	**Supernatant**	**Pellet (EXO)**	**Supernatant**	**Pellet (EXO)**	**Supernatant**	**Pellet (EXO)**
miR-302a	ND	ND	36.43±0.21	26.47±0.35	ND	ND	35.67±0.25	24.09±0.22
miR-302b	ND	ND	35.82±0.13	25.23±0.10	ND	ND	34.72±0.18	25.22±0.55
miR-302c	ND	ND	34.12±0.43	25.29±0.05	ND	ND	34.22±0.23	23.23±0.11
miR-302d	ND	ND	35.46±0.31	24.34±0.025	ND	ND	34.64±0.22	23.30±0.01
miR-367	ND	ND	34.29±0.37	28.77±0.025	ND	ND	34.40±0.17	27.98±0.14

The values correspond to the number of cycles obtained using equal amount of RNA extract in three separate experiments
