# Supplementary figures and images for: Cell-based therapy using miR-302-367 expressing cells represses glioblastoma growth
Source: Cell Death Dis. 2017 Mar 30;8(3):e2713–. doi: 10.1038/cddis.2017.117 (PMC5386523; doi:10.1038/cddis.2017.117)

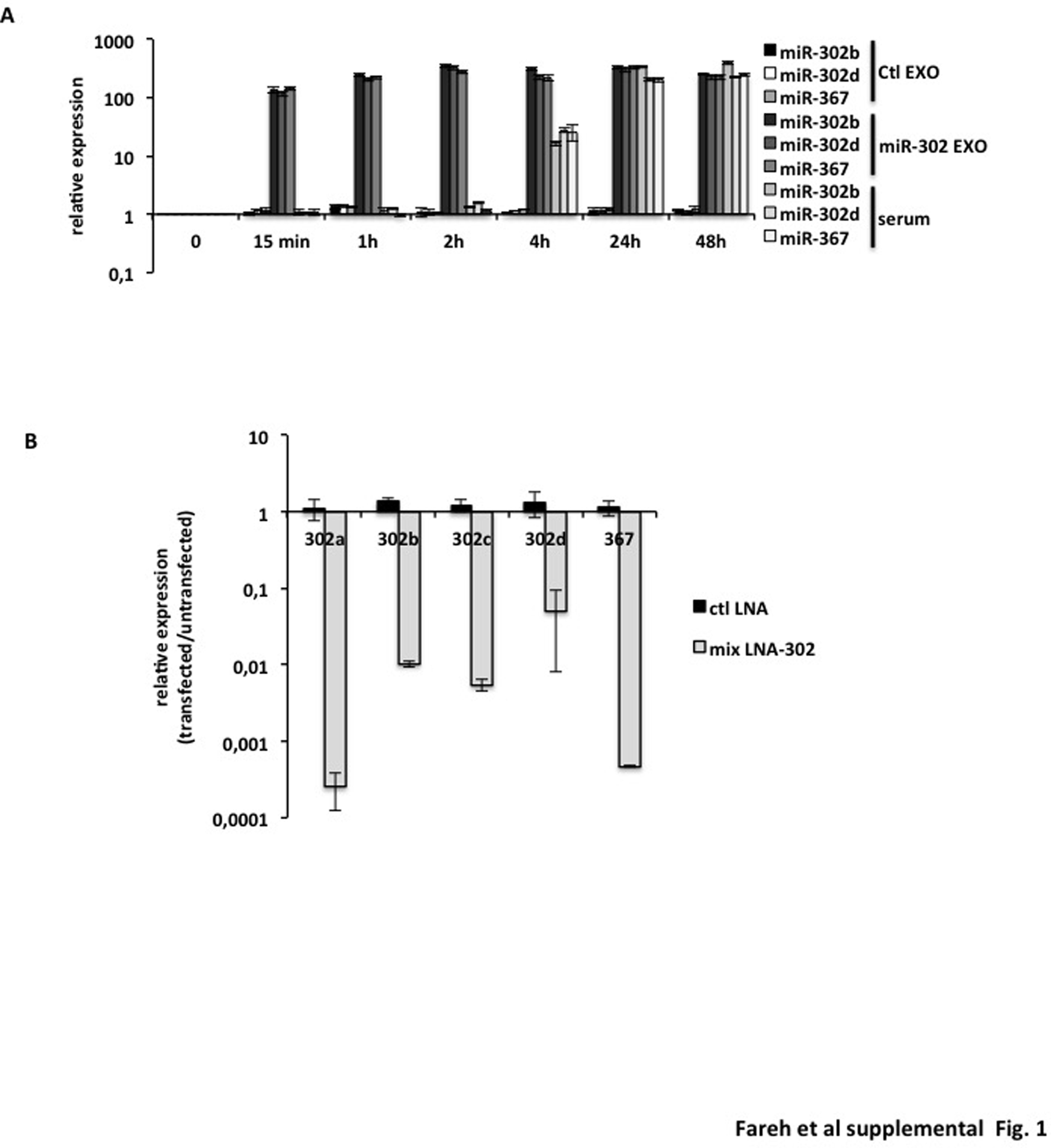

Supplement: Supplementary Figure 1 [file cddis2017117x1.tif]

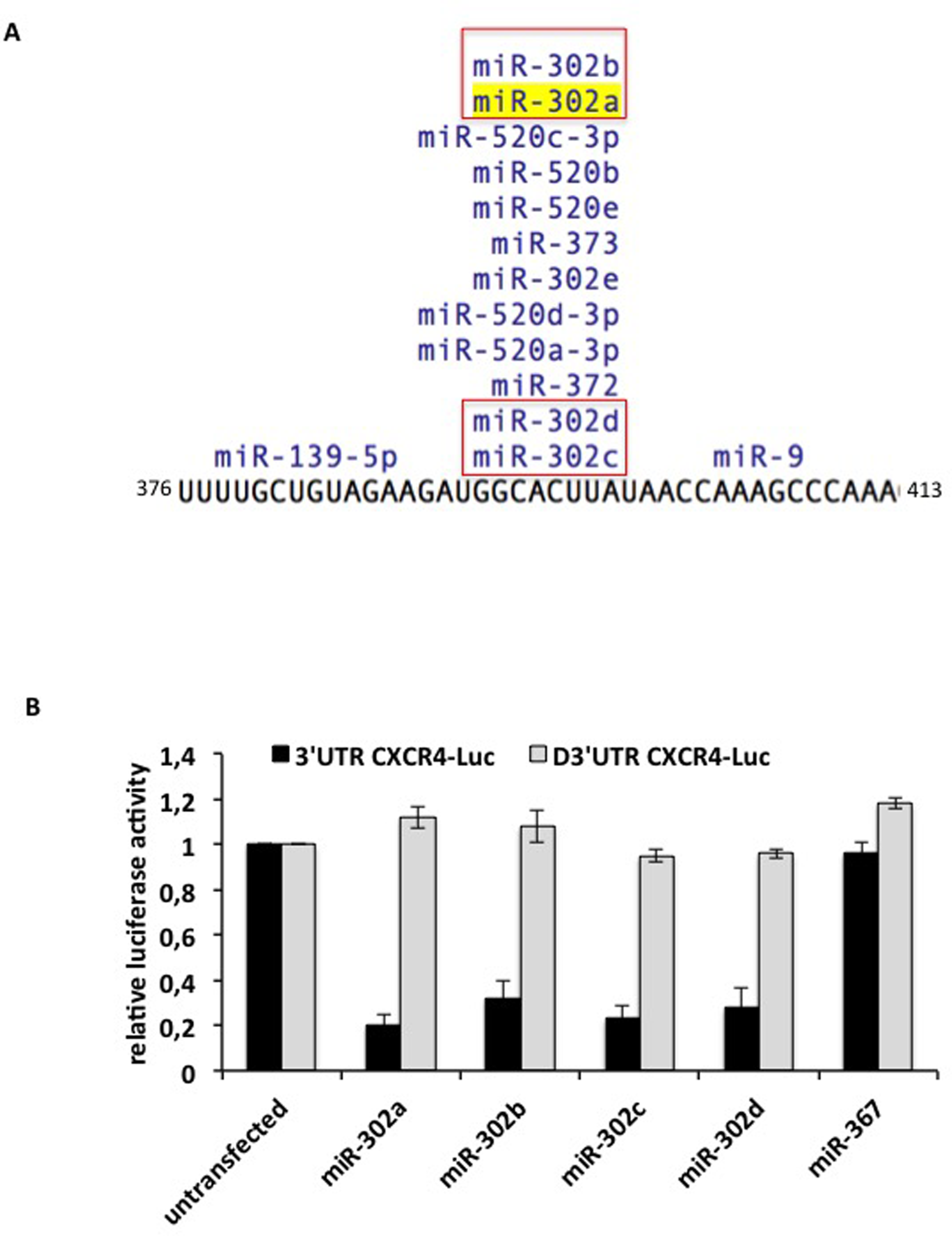

Supplement: Supplementary Figure 2 [file cddis2017117x2.tif]
